# Eighteen years’ experience of traumatic subclavian vascular injury in a tertiary referral trauma center

**DOI:** 10.1007/s00068-018-01070-5

**Published:** 2019-01-09

**Authors:** Hao-Wei Kou, Chien-Hung Liao, Jen-Fu Huang, Chih-Po Hsu, Shang-Yu Wang, Chun-Hsiang Ou Yang, Shih-Ching Kang, Yu-Pao Hsu, Chi-Hsun Hsieh, I-Ming Kuo

**Affiliations:** Division of Trauma and Emergency Surgery, Department of Surgery, Chang Gung Memorial Hospital, Chang Gung University, No.5, Fuxing St., Guishan Dist., Linkou, Taoyuan, 333 Taiwan

**Keywords:** Traumatic subclavian vascular injury, Blunt thoracic trauma, Head injury, Clavicular fracture, Scapular fracture

## Abstract

**Purpose:**

Traumatic subclavian vascular injury (TSVI) is rare but often fatal. The precise diagnosis of TSVI remains challenging mainly because of its occult nature, less typical presentations, and being overlooked in the presence of polytrauma. Compared to penetrating injuries, it is even more difficult to identify TSVI in patients who have blunt injuries and no visible bleeding. The risk factors associated with TSVI in patients with thoracic trauma are unclear. The aims of this study were to identify risk factors for TSVI in a cohort of patients with thoracic vascular injuries and to report outcomes after clinical treatment.

**Methods:**

From January 2009 to June 2017, 39586 patients were admitted to our hospital (a level I trauma center) due to trauma, and 136 patients with thoracic vascular injury were enrolled in this study. We retrospectively reviewed data from medical records including demographic characteristics, injury scoring systems (RTS, ISS, NISS, TRISS and AIS), management and outcomes. Patients were further divided into the TSVI group (patients with TSVI) and the non-TSVI group (patients with thoracic vascular injuries other than TSVI). Univariate and multivariate analyses were used to identify independent risk factors.

**Results:**

The enrolled 136 patients suffered mostly from blunt trauma (89.0%) and 22 of them had TSVI. When compared to the non-TSVI group, the TSVI group had lower Glasgow Coma Scale (GCS) scores (*p* = 0.002; especially GCS ≤ 12), less concurrent abdominal injury (*p* < 0.001), lower Injury Severity Scales (ISS) (*p* = 0.007) and New Injury Severity Scales (NISS) (*p* < 0.002) but had higher Abbreviated Injury Scales (AIS) of the head ≥ 3 (*p* = 0.009) and rates of clavicular or scapular fractures (*p* = 0.013). No difference was detected between the two groups with regard to age, gender, trauma mechanism, vital signs on arrival, or rate of facial and extremities injury. In multivariate regression analyses, GCS ≤ 12, AIS of the head ≥ 3 and the presence of clavicular or scapular fractures were independent risk factors for TSVI (*p* = 0.026, *p* = 0.043 and *p* = 0.005, respectively) after adjustment for confounding factors. Open and endovascular repair were two surgical procedures utilized for these TSVI patients with an overall mortality rate of 18.2%. No difference was found between these groups with regard to mortality rate and the length of ICU stay, but the patients in the TSVI group had a shorter length of hospital stay.

**Conclusions:**

Our results suggest that GCS ≤ 12, AIS of the head ≥ 3 and the presence of clavicular or scapular fractures were independent risk factors for TSVI in patients with thoracic vascular injuries. For patients with thoracic trauma, TSVI should be considered for prompt management when patients exhibit concurrent injuries to the head, clavicle or scapula.

## Background

Traumatic thoracic vascular injuries are often lethal in patients with thoracic trauma. Among these injuries, traumatic subclavian vascular injury (TSVI) is relatively uncommon because of protection by bony and muscular structures [1–7]. Although the incidence is rare, TSVI can be fatal with a mortality rate as high as 30% [1, 2, 8, 9]. Because of its rarity, this kind of vascular injury is not well characterized and this results in a lack of standardization in management [1, 6, 7]. Delay in diagnosis and overlooked injuries are known to profoundly influence the outcome in these patients [1, 10]. The precise diagnosis of TSVI remains a challenge for clinicians.

These patients often have complicated polytraumas which draw the physician’s attention away from the potentiality of TSVI. Most of the reported patients with TSVI had various penetrating injuries [1, 6, 7]. On the other hand, it is even more difficult to identify TSVI in a patient with blunt trauma who may have no source of visible bleeding [11]. Subclavian vessels are located in the region between the mediastinum and upper extremity so that this type of injury may present with distinctive signs. Some studies have noted that the presence of certain signs (e.g. decreasing pulse in the upper limbs) or associated injuries (e.g. head injuries) should cause physicians to think about the possibility of TSVI, because a prompt diagnosis necessitates a high index of suspicion [1, 6, 7, 10, 12–16]. These reports described findings based on observations of case series, and no comparison and analysis of data was available. The purpose of this study was to identify the risk factors for TSVI by comparing these patients with those with other thoracic vascular injuries. The management and outcomes were also described and analysed.

## Materials and methods

### Patient population

From January 2009 to June 2017, a total of 39,586 patients were admitted to the emergency department and hospitalized in Linkou Chang Gung Memorial Hospital, a level I trauma center, due to trauma. Patients with thoracic vascular injury were identified and extracted from the institution’s trauma registration database. Those with injuries associated with vascular grafts were excluded, and 136 patients were enrolled in this study. These patients were evaluated and treated based on the advanced trauma life support (ATLS) guidelines. A whole body computerized tomography (CT) scan was provided for the patients with major trauma, or computerized tomography angiography (CTA) of the chest for patients with suspected thoracic vascular injuries. The diagnosis was confirmed either by surgical exploration or by imaging studies.

### Data collection and statistical analysis

We retrospectively reviewed data including demographic characteristics, trauma mechanism, trauma scoring systems [Revised Trauma Score (RTS), Abbreviated Injury Score (AIS), Injury Severity Score (ISS), New Injury Severity Score (NISS) and The Trauma and Injury Severity Score (TRISS)], vital signs and level of consciousness on arrival, laboratory exams, imaging studies and subsequent management (including open surgical repair, endovascular repair, embolization and observation). The length of hospitalization, the length of the intensive care unit (ICU) stay and mortality were also noted. These patients were divided into the TSVI and non-TSVI groups for risk analyses. Those with concurrent TSVI and other vascular injuries were included in the TSVI group.

Continuous data are presented as mean ± SEM. The Kolmogorov–Smirnov test was used to check the distribution of the continuous variables. Student’s *t* test and the Mann–Whitney *U* test were employed to compare quantitative variables between the two groups. Pearson’s *χ*^2^ test or Fisher’s exact test were used to compare categorical variables. *p* values < 0.05 were considered statistically significant. Factors with statistical significance disclosed by univariate analyses were included in the multivariate analysis using logistic regression. All statistical analyses were performed using SPSS v17.0 (SPSS Inc., Chicago, IL, USA).

### Ethics approval and consent to participate

This retrospective analysis was approved by the Chang Gung Medical Foundation Institutional Review Board (201801069B0). The Chang Gung Medical Foundation Institutional Review Board determined that written informed consent from the patients or their families was not necessary for this kind of retrospective study.

## Results

### Demographic characteristics

**Fig. 1:**
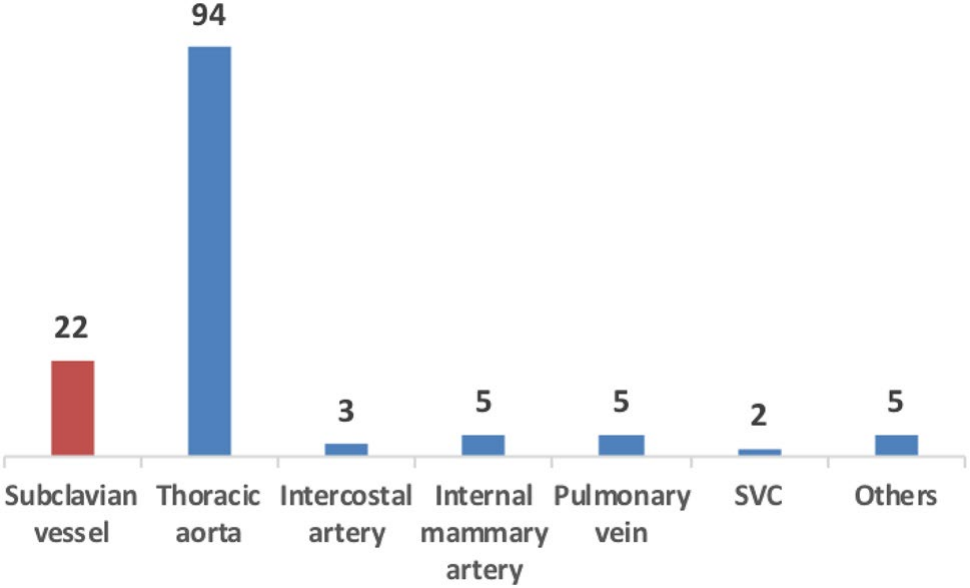
Types of thoracic vascular injuries in our patient group. *SVC* superior vena cava, *Others* inclusive of cephalic vein (1), inferior phrenic artery (1), and unknown vascular injury (3)

The mean age of these patients was 42.8 ± 1.49 years. There were 115 males (84.6%) and 21 females (15.4%) in the group. Ninety-six (70.6%) were referred from other hospitals. A total of 121 (89.0%) suffered from blunt trauma, while the others (11.0%) had penetrating injuries. Motor vehicle collisions accounted for 70.6% of these injuries. Other causes included falling from a height (16.2%), stabbing (6.6%), crushing (5.2%), gunshot (0.7%) and blast injuries (0.7%). In total, 22 patients (16.2%) had TSVI and 20 of these were arterial injuries. One had concurrent injuries of the subclavian artery and pulmonary vein. Figure 1 shows the distribution of various thoracic vascular injuries in the study.

Clinical characteristics and demographics were compared between these two groups (Table 1). There were no differences in age, gender, mechanism of trauma, or vital signs on arrival between the two study groups. The patients with TSVI had lower Glasgow Coma Scale (GCS) scores (*p* = 0.002), especially GCS ≤ 12 (*p* = 0.015) but lower ISS (23.0 vs. 29.7, *p* = 0.007) and NISS (28.5 vs. 37.6, *p* = 0.002). AIS of the head was significantly higher in the TSVI group than in the non-TSVI group (*p* = 0.042), especially in patients with AIS ≥ 3 (*p* = 0.009). The patients with TSVI had fewer concurrent abdominal injuries (*p* < 0.001) but more clavicular or scapular fractures (*p* = 0.013). There was no difference in the incidence of injuries to the face or limbs (Table 2). The results of multivariate analysis are shown in Table 3. GCS less than 12, AIS of the head ≥ 3 and the presence of clavicular or scapular fractures were independent risk factors for TSVI (*p* = 0.012, 0.011 and 0.015, respectively) after adjustment for confounding factors. The presence of concurrent abdominal injury was an independent factor associated with thoracic vascular injuries rather than injury to subclavian vessels (*p* = 0.003).

**Table 1:** Clinical characteristics and demographics

	TSVI group (*n* = 22)	Non-TSVI group (*n* = 114)	*p* value
Age	36.0 ± 3.50	44.1 ± 1.62	0.052
Gender			1.000
Male	19 (86.4)	96 (84.2)	
Female	3 (13.6)	18 (15.8)	
Mechanism			0.097
Vehicle accident	15 (68.2)	81 (71.1)	
Falling accident	1 (4.5)	21 (18.4)	
Penetrating injury	4 (18.2)	7 (6.1)	
Crushing injury	2 (9.1)	5 (4.4)	
Blunt injury			0.265
Yes	18 (81.8)	103 (90.4)	
No	4 (18.2)	11 (9.6)	
Vital signs at triage			
Body temperature (°C)	36.2 ± 0.31	35.9 ± 0.33	0.644
Pulse rate (beats/min)	100.2 ± 5.03	100.7 ± 3.01	0.948
MAP (mmHg)	88.1 ± 5.63	83.4 ± 3.06	0.530
SBP < 90 (mmHg)	4 (18.2)	31 (27.2)	0.376
Respiratory rate (breaths/min)	19.2 ± 0.62	21.3 ± 0.70	0.200
GCS			0.002
3–8	7 (31.8)	33 (28.9)	
9–12	7 (31.8)	8 (7.0)	
13–15	8 (36.4)	73 (64.0)	
GCS ≤ 12	14 (63.6)	41 (36.0)	0.015

Continuous data expressed as mean ± SEM (standard error of the mean) and categorical data as number (%)

*TSVI* traumatic subclavian vascular injury, *MAP* mean arterial pressure, *SBP* systolic blood pressure, *GCS* Glasgow Coma Scale

**Table 2:** The details of associated injuries and prognosis

	TSVI group (*n* = 22)	Non-TSVI group (*n* = 114)	*p* value
RTS	6.41 ± 0.32	6.49 ± 0.19	0.362
ISS	23.0 ± 2.2	29.7 ± 0.9	0.007
NISS	28.5 ± 2.4	37.6 ± 1.1	0.002
TRISS	0.856 ± 0.040	0.759 ± 0.027	0.149
AIS-head			0.042
0	7 (31.8)	78 (68.4)	
1	1 (4.5)	3 (2.6)	
2	3 (13.6)	7 (6.1)	
3	5 (22.7)	11 (9.6)	
4	5 (22.7)	10 (8.8)	
5	1 (4.5)	5 (4.4)	
AIS-head ≥ 3	11 (50.0)	6 (22.8)	0.009
AIS-face			0.110
0	13 (59.1)	91 (79.8)	
1	4 (18.2)	10 (8.8)	
2	5 (22.7)	13 (11.4)	
Concurrent abdominal injury			< 0.001
Yes	1 (4.5)	57 (50.0)	
No	21 (95.5)	57 (50.0)	
Concurrent extremity injury			0.315
Yes	12 (54.5)	75 (65.8)	
No	10 (45.5)	39 (34.2)	
Clavicle or scapula fracture	8 (36.4)	15 (13.2)	0.013
Hospital stay (days)	12.2 ± 1.79	18.8 ± 1.27	0.025
ICU stay (days)	6.1 ± 0.82	7.5 ± 0.74	0.945
Mortality	4 (18.2)	22 (19.3)	1.000
Mortality < 24 h	1 (4.5)	18 (15.8)	0.306

*RTS* Revised Trauma Score, *ISS* Injury Severity Score, *NISS* New Injury Severity Score, *TRISS* Trauma And Injury Severity Score, *AIS* Abbreviated Injury Score, *ICU* intensive care unit

**Table 3:** Multivariate analysis of risk factors for subclavian vessel injuries

Variables	OR	95% CI	*p* value
GCS ≤ 12	6.276	1.658–26.130	0.012
Clavicle or scapula fracture	6.766	1.443–31.720	0.015
AIS score-head ≥ 3	7.417	1.071–34.469	0.011
Concurrent abdominal injury	0.018	0.001–0.259	0.003
ISS	0.892	0.7873–1.029	0.117
NISS	0.942	0.853–1.041	0.243

*OR* odds ratio after adjustment for confounding factors

### Managements and outcomes

The types of TSVI in our study included pseudoaneurysm (*n* = 8), laceration (*n* = 3 artery and 2 vein), dissection (*n* = 2), transection (*n* = 2), occlusion (*n* = 1) and unclassifiable (*n* = 4). Open and endovascular repairs were performed in six and seven patients, respectively. Two patients received transcatheter arterial embolization (TAE). Although two patients expired before definite treatment, five were managed uneventfully under conservative observation (Table 4). Those who received endovascular repair all survived, but two patients with subclavian arterial injuries died after open repair. The overall mortality rate was 18.2% (*n* = 4) and all of these were due to arterial injuries. Head injury was the major cause of death for three of the four patients and the other died of hemorrhage from the injured subclavian artery. There was no difference between the TSVI group and the non-TSVI group in overall mortality and early mortality (died within 24 h) rates (18.2% vs. 19.3%, *p* = 1 and 4.5% vs. 15.8%, *p* = 0.306, respectively). Nevertheless, there was a significant difference in the length of hospital stay (12.2 ± 1.79 vs. 18.8 ± 1.27 days, *p* = 0.025), but not in the length of ICU stay (6.1 ± 0.82 vs. 7.5 ± 0.74, *p* = 0.945), between the two groups.

**Table 4:** The type of subclavian vascular injury and its management

Type	Number(s)	Treatment
Artery		
Pseudoaneurysm*	8	Endovascular repair, *n* = 5; embolization, *n* = 2
Laceration	3	Open repair, *n* = 3
Dissection	2	Observation, *n* = 2
Transection	2	Endovascular repair, *n* = 1; observation, *n* = 1
Occlusion	1	Endovascular repair, *n* = 1
Unclassifiable*	4	Open repair, *n* = 1; observation, *n* = 2
Vein		
Laceration	2	Open repair, *n* = 2

*One patient expired before treatment

## Discussion

The incidence of TSVI remains unclear. Rulliat et al. [17] reported that the incidence rate of subclavian arterial rupture among 1181 thoracic injuries was about 0.4%. Some studies [1, 6] have reported that subclavian vascular injuries represented only 3–9% of all vascular trauma, and most of the cases with TSVI (75–92.5%) resulted from penetrating injury by firearms or knives [1, 6, 7, 18]. In the current study, we found the incidence rate of TSVI to be 0.05% in all trauma patients hospitalized, and this type of injury represented 16.2% of thoracic vascular injuries. The majority of TSVI in our study was from blunt thoracic trauma (81.8%) caused by motor vehicle accidents. The population of our patients was far different from those of previous studies.

The clinical diagnosis of TSVI is still challenging because of its various presentations. The most frequent manifestation is a sign resulting from arterial occlusion, such as diminished or absent pulses in the upper limbs [12]. Sturm and Cicero described five criteria for suspected subclavian vascular injury, including fractures of the first rib, diminished or absent radial pulses, palpable supraclavicular hematoma, a widened mediastinum or a hematoma over the area of the subclavian artery demonstrated by chest roentgenograms and brachial plexus palsy [13]. Some studies reported, however, that only 20% of patients had “hard signs”, and a pulse deficit was found in only 32–59% of patients with TSVI [1, 6, 12]. It is worth noting that these reports focused primarily on penetrating injuries. In our study, only 6 of 22 patients (27.3%) had hard signs, including 3 with peri-clavicular hematoma, 2 with diminished radial pulses and 1 with massive hemothorax. Most of our patients had blunt injuries which resulted in vascular injury rather than direct perforations [14, 18]. As a result, the leading type of vascular injury in our patient cohort was pseudoaneurysm formation so that a lower incidence of obvious pulse deficit could be expected. Our findings indicated that blunt trauma-related TSVI was difficult to identify by clinical presentation.

The precise diagnosis of TSVI remains difficult in the presence of polytrauma [1]. Investigation of the risk factors associated with TSVI among patients with thoracic vascular injuries has been of great interest [1, 6, 7, 10, 14–16, 19]. In this study, TSVI patients exhibited several clinical features distinctive from those with other thoracic vascular injuries. We found that GCS ≤ 12, AIS of the head ≥ 3 and the presence of clavicular or scapular fractures were associated with an increase, while the presence of concurrent abdominal injury was associated with a reduction, of TSVI. These comparative analyses provided valuable indications for the early diagnosis of TSVI in patients with thoracic injuries. The two risk factors of GCS ≤ 12 and AIS of the head ≥ 3, strongly suggested that TSVIs were complicated by head injuries. Two case-series studies have reported that head injuries were one of the most common injuries associated with TSVIs [10, 16]. Head injuries have been found to be one of the major causes of death in patients with subclavian artery injuries [7]. To the best of our knowledge, this was the first study to provide compelling evidence for the relationship between head injury and TSVI. We identified the presence of clavicular or scapular fractures as a risk factor for TSVI as well. The incidence of vascular injury with peri-clavicular trauma was about 5.5–14% [1]. Some studies have reported that TSVI was one of the more common injuries associated with fractures of the clavicle [6, 14, 18], while a few studies have addressed the association of scapular fracture with TSVI [15, 19]. In this study, clavicular fracture occurred in 27.3% of our patients and there were two cases with scapular fractures. All suffered from blunt thoracic trauma due to motor vehicle accidents. Generally, these findings suggest that high-energy, blunt injuries to the head or upper torso may potentially result in TSVI. This is also compatible with our finding that patients with concurrent abdominal injury had fewer injuries to subclavian vessels.

ISS and NISS in the TSVI group were significantly lower than those in the non-TSVI group, but their RTS, TRISS and mortality rates were similar. Previous studies revealed that the mortality rate for subclavian artery injuries ranged from 3 to 33% [1, 2, 5–7, 20, 21]. Four of our 22 patients (18.2%) with TSVI died, three of them from head injuries. There was no difference between the two groups with regard to the length of ICU stay. This discrepancy shows the impact of other associated injuries on the clinical course. Delay in recognition of this type of vascular injury might result in inappropriate management.

For patients with a traumatic subclavian arterial injury, endovascular repair has been advocated [12, 22–24]. Traumatic subclavian vein injury can be also treated successfully by open repair or ligation [20]. In this study, endovascular and open repair were both effective for TSVI. Although endovascular management is less invasive and more technique-dependent, the benefits and timing of these minimally invasive procedures still need further investigation.

There were some limitations to the current study. First, this was a retrospective study with relatively few case numbers, and the findings cannot be generalized to patients with penetrating injuries. The second limitation was the lack of long-term follow-up. Further studies with a larger sample size and evaluation of disability are needed.

## Conclusions

Our results suggest that GCS ≤ 12, AIS of the head ≥ 3 and the presence of clavicular or scapular fractures were independent risk factors for TSVI in patients with thoracic vascular injuries. Those with concurrent thoracic and abdominal injuries had fewer injuries to subclavian vessels. For patients with thoracic trauma, TSVI should be considered for prompt management when patients exhibit concurrent injuries to the head, clavicle or scapula. Although endovascular repair is the current trend in management, the benefits still need further investigation.
